# Partially hydrolyzed guar gum supplement reduces high-fat diet increased blood lipids and oxidative stress and ameliorates FeCl_3_-induced acute arterial injury in hamsters

**DOI:** 10.1186/1423-0127-16-15

**Published:** 2009-02-02

**Authors:** Dar-Chih Kuo, Shih-Ping Hsu, Chiang-Ting Chien

**Affiliations:** 1 Department of Cardiovascular Surgery, Kuang-Tien General Hospital, Taichung, Taiwan; 2 Department of Internal Medicine, Far-Eastern Memory Hospital, Taipei, Taiwan; 3 Institute of Clinical Research, National Taiwan University Hospital and National Taiwan University College of Medicine, Taipei, Taiwan; 4 Department of Medical Research, National Taiwan University Hospital and National Taiwan University College of Medicine, Taipei, Taiwan

## Abstract

Increased reactive oxygen species (ROS) and hyperlipidemia can promote arterial thrombus. We evaluated the potential of a partially hydrolyzed guar gum (PHGG) as dietary fiber on lipid profiles and FeCl_3_-induced arterial thrombosis in the high fat-diet fed hamsters. Our *in vitro *results found that PHGG is efficient to scavenge O_2_^-^•, H_2_O_2_, and HOCl. High fat-diet increased plasma triglyceride, total cholesterol, LDL, VLDL, methylguanidine and dityrosine level and accelerated FeCl_3_-induced arterial thrombosis formation (from 463 ± 51 to 303 ± 45 sec). Low dose PHGG supplement significantly decreased the total cholesterol, LDL, methylguanidine and dityrosine level and delayed the time for arterial thrombosis formation (528 ± 75 sec). High dose PHGG supplement decreased the level in triglyceride, total cholesterol, LDL and VLDL and further delayed the time for arterial thrombus (671 ± 36 sec). The increased Bax protein and decreased Bcl-2 and HSP-70 protein expression was found in the carotid and femoral arteries of high fat-diet hamsters. Low and high dose of PHGG supplement decreased Bax expression and increased Bcl-2 and HSP-70 protein expression. We found that FeCl_3 _significantly enhanced intercellular adhesion molecule-1 and 4-hydroxynonenal expression in the endothelial site of damaged artery after 150-sec FeCl_3 _stimulation. PHGG supplement decreased the endothelial ICAM-1 and 4-hydroxynonenal expression after 150-sec FeCl_3 _stimulation. Based on these results, we conclude that PHGG supplement can increase antioxidant protein expression and thus decrease oxidative stress induced arterial injury.

## Background

It is generally accepted that excess reactive oxygen species (ROS) production can cause oxidation of biological macromolecules and produce damage to biological systems [1-5]. The major ROS like O_2_^-.^, H_2_O_2 _and HOCl, which can produce lipid peroxidation products, malonealdehyde and phosphatidylcholine hydroperoxide [3,6] and protein oxidation, dityrosine and methylguanidine, as indirect indicators of ROS and/or free-radical activity [6,7]. Locally enhanced vascular production of ROS decreases the bioavailability of nitric oxide, impairs vascular relaxation, and promotes leukocytes and platelet adhesion/aggregation [8]. On the other hand, increased levels of cholesterol or lipid profiles in the plasma is a status called hyperlipidemia, which can lead to serious problems such as coronary heart disease and other cardiovascular diseases [9]. There is evidence that adhesion of monocytes to the endothelium and transformation of macrophages into foam cells, plaque stability, vasomotor function, platelet aggregation and tendency to thrombosis lead to the development of atherosclerosis and coronary artery disease and can be modified by antioxidants [10,11]. Therefore, a dynamic interaction exists between endothelial and thrombosis/atherogenesis, both of which may be influenced by oxidative stress and lipid profiles. Adhesion of platelet and leukocytes into the vascular endothelial cell by the increased expression of intercellular adhesion molecule-1 (ICAM-1) is a crucial step in thrombosis/atherogenesis [12]. Suppressed expression of ICAM-1 was associated with reduced adherence of monocytes, lymphocytes and platelets to oxidized LDL stimulated endothelial cells [13,12]. The defense of vascular endothelium against the damage caused by ROS and dyslipidemia is probably an important factor for reduction of thrombotic and atherosclerotic attack [10]. Vascular wall protection can be achieved by preventive attachment to the vascular wall of antioxidants and elimination/neutralization of toxic products after their disproportioning [14,15].

**Figure 1:**
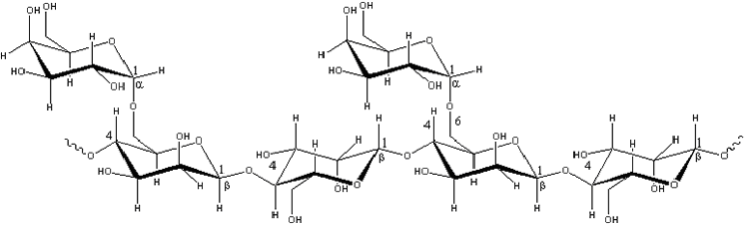
**Chemical structure of partially hydrolysed guar gum (PHGG). The chemical structure of PHGG: mannose: galactose = 2:1**.

Lipid-lowering strategy may have a beneficial role in normalizing vascular function and greatly decreasing the frequency of clinical events associated with atherosclerosis, combined with the ability of antioxidants to alleviate vasomotor disturbances in hypercholesterolemia and to slow the progression of atherosclerosis [3]. There is an increase in interest in finding a naturally antioxidant plant-based compound, which could reduce the increased lipid levels in plasma. Guar gum, a galactomannan derived from the cluster bean of Cyamopsis tetragonolobus which is cultivated in India, Pakistan, and the United States, and its enzymatic hydrolysate (partially hydrolysed guar gum, PHGG) shown in Figure 1 have been shown to decrease levels of serum cholesterol and lipids and postprandial blood glucose [16]. The present study was carried out in hamsters adapted to high-fat diet to compare the lipid-lowering effectiveness of PHGG and to examine the impact of PHGG supplement on FeCl_3_-induced acute arterial injury in the animals. The antioxidant activity and mechanism in PHGG on FeCl_3 _induced arterial thrombosis was also evaluated in the present study.

## Methods and materials

### Changes of lucigenin- and luminol-enhanced chemiluminescence counts (CL)

We evaluated the antioxidant activity of PHGG on xanthine (0.75 mg kg^-1^, dissolved in 0.01 N NaOH) and xanthine oxidase (24.8 mU kg^-1^) enhanced O_2_^-.^, 0.03% H_2_O_2 _induced H_2_O_2 _activity, and 0.03% HOCl induced HOCl activity. Xanthine and xanthine oxidase were purchased from Sigma, USA. ROS levels were measured by a CL analyzing system (CLD-110, Tohoku Electronic Industrial, Sendai, Japan) as described previously (7). The system contains a photon detector (Model CLD-110), CL counter (Model CLC-10), water circulator (Model CH-200) and 32 bit IBM personal computer system. A cooler circulator is connected to the model CLD-110 photon detector to keep the temperature at 5°C. The CLD-110 model is extremely sensitive; it is able to detect as little as 10^-15 ^W of radiant energy. The CL was measured in an absolutely dark chamber of the CL analyzing system. We demonstrated that using CL-emitting substance lucigenin (*N*, *N*'-dimethyldiacridinium, Sigma, St. Louis, MO, USA) for O_2_^-. ^or luminol (5-amino-2,3-dihydro-1,4-phthalazinedione, Sigma, St. Louis, MO, USA) for H_2_O_2 _or HOCl (hydrogen peroxide, sodium hypochlorite, Sigma, St. Louis, MO, USA) to enhance the CL counts provided similar data to those reported in our previous study [1,3]. The lucigenin-enhanced CL method provides a reliable assay for superoxide. After 100 s, 1.0 ml of 0.1 mM lucigenin in PBS (pH = 7.4) was mixed with the tested sample. The CL in the tested sample was measured continuously for a total of 600 s. The assay was performed in duplicate for each sample, and the results are expressed as CL counts (10 s)^-1^. The total amount of CL in 600 s was calculated by integrating the area under the curve. The mean ± S.E.M. CL level for each sample was calculated.

### Animals

Golden Syrian hamster (*Mesocricetus auratus*), male 12-week old, 110–120 g body weight were used and were housed at the Experimental Animal Center, National Taiwan University. A group of eight animals (four/cage) were kept in controlled conditions, temperature 25–26°C, relative humidity 60–80% and 12/12 h light/dark cycle (light from 8.00 a.m. to 8.00 p.m.). Food and water were provided ad libitum. The animals with identification marks were acclimatized for 7 days before experiments. All surgical and experimental procedures were approved by National Taiwan University College of Medicine and College of Public Health Institutional Animal Care and Use Committee and are in accordance with the guidelines of the National Science Council of Republic of China (NSC 1997).

The high-fat diet (HF) was prepared by mixing the normal pellet diet with groundnut oil, cholesterol (Sigma), deoxycholesterol (Sigma), and fructose (Sigma) in a ratio of 610 g:300 ml:5 g:5 g:100 g, respectively, to a final weight of 1.0 kg. This homogenous soft cake was molded in the shape of pellets of about 3 g each. The average caloric intake is estimated from the control diet and high fat diet, respectively. Animals of all the groups were fed with HF (10 g/animal) for 4 days before PHGG treatment. The animals had free access to HF and water for 28 days (days 1–28). Subsequently they were randomly divided into four groups (n = 11 each); Group 1: animals received normal powdered rodent diet (control) (purchased from LabDiet, Nutrition International Inc. USA); Group 2:, animals received HF; Group 3 and 4: 0.25 mg (HFLP) and 1.0 mg PHGG (HFHP) were administered in the animals received HF. PHGG was supplied by Taiyo Kagaku Co., Ltd., Japan.

### Collection of blood samples and biochemical analysis from plasma

The collection of blood was made on day 28, 2 h after the last PHGG administration. Hamsters (non-fasted) were anesthetized by sodium pentobarbital (50 mg/ml in normal saline), injected i.p. (1 ml/kg body weight). Blood was drawn from the jugular vein and collected in EDTA-coated tubes (3 mg/ml) by centrifugation at 1,500 × g for 5 min at 4°C for the estimation of plasma total cholesterol, triglycerides, and high-density lipoprotein (HDL), LDL, very low-density lipoprotein (VLDL), and cholesterol values were measured [3]. The measurement of lipoprotein(a) [Lp(a)] was performed with a commercial LPA kit (#465360, Beckman Coulter-Array System, Denmark). The analysis of plasma biochemical parameters was performed on an auto-analyzer of Beckman Coulter model, 'Synchron-CX-5 Clinical System' by standard enzymatic methods, and assay kits were purchased from Beckman Coulter International USA.

### FeCl_3 _induced acute arterial thrombosis

All the animals were anesthetized by subcutaneous injection of 1.2 g/kg urethane (Sigma-Aldrich Inc., St. Louis, MO, USA). A PE-50 catheter was catheterized in the femoral vein for intravenous administration of tested saline by an infusion pump (Infors AG, CH-4103, Bottmingen, Switzerland) at a rate of 1.2 ml/min. After arterial isolation, transonic flow probes (Probe# 0.5VBB517, Transonic Systems Inc., Ithaca, NY, USA) for carotid arterial blood flow measurement were applied and were displayed on a small animal blood flow meter (Model 206, Transonic Systems Inc., Ithaca, NY, USA). All the blood flow signals were continuously recorded by an ADI system (PowerLab/16S, ADI Instruments, Pty Ltd, Castle Hill, Australia). The carotid artery was injured as previously described [11] with slight modification. A filter paper (1 mm × 2 mm) soaked with 30% FeCl_3 _solution (Ferric chloride, Sigma, St. Louis, MO, USA), was applied to the artery for 3 minutes and the cavity was filled with saline immediately [11]. The FeCl_3 _solution easily diffused into the arterial tissue induced a Fenton reaction mediated endothelial injury (impairment of nitric oxide release) and subsequently induce vascular constriction to reduce arterial blood flow within minutes [11]. The flow rate was monitored and recorded and the time to occlusion (TTO, arterial blood flow decreases to zero) was determined.

### 4-HNE products and ICAM-1 expression in the carotid artery

We considered that the high levels of FeCl_3 _via Fenton reaction enhanced ROS might promote the expression of ICAM-1 and the accumulation of lipid peroxides, 4-hydroxynonenal (4-HNE) in the endothelium for induction of thrombosis within minutes. We evaluated the expression of 4-HNE and ICAM-1 after 150 sec of FeCl_3 _stimulation in four groups of hamsters (n = 3 in each group). 4-hydroxynonenal (4-HNE) [5] and ICAM-1 expression [17] in the OCT-embedded sections of vascular rings were immunostained. On the day of staining, we removed the frozen slides from -20°C and thawed at 25°C for 30 min. After rinsing in 0.2 mol/L of phosphate buffer saline (PBS), the sections were incubated in 3% hydrogen peroxide in 0.2 mol/L of PBS for 30 min, followed by incubation in blocking buffer (5% goat serum, 0.25% Triton-X, 1% dry milk in 0.2 mol/L PBS) for 1 h, followed by the exposure to specific primary antibody. The 30-μm cross sections were stained immunohistochemically for presence of in vivo markers of lipid peroxidation, 4-HNE-protein adducts, by incubation with a polyclonal antibody (Alpha Diagnostic International; San Antonio, TX, USA) and antibody raised against ICAM-1 (R&D Systems, Minneapolis, MN, USA) diluted at 1:50 overnight. The following day, sections were incubated for 2 h at 25°C with biotinylated goat-anti-rabbit secondary antibody (1:200, vector ABC-AP kit; vector Labs, Burlingame, CA, USA). The sections were stained with an avidin-biotinylated horseradish-peroxidase procedure using a commercially available kit (ABC Elite; Vector Laboratories). Finally, the color was developed by the 3'-Amino-9-ethylcarbazole substrate (Vector Laboratories). The 4-HNE and ICAM-1 stains were photographed on an Leica microscope (model DMRD, Leica Microsystems Wetzlar, Wetzlar, Germany). The expressed percentage of endothelial 4-HNE and ICAM-1 expression was analyzed by the curvature of circular stain/360° × 100%. The percentage of 4-HNE and ICAM-1 expression in the vascular ring was calculated as following formula.

### Immunoblot analysis of antioxidant- and anti-apoptosis-related proteins

Antibodies raised against mouse anti-human Bcl-2 (Transduction, Bluegrass-Lexington, KY, USA), Bax (Chemicon, Temecula, CA, USA) and primary polyclonal rabbit antihuman HSP-70 (Transduction, Bluegrass-Lexington, KY, USA) antibodies were diluted at 1:400. (BioVision, Mountain View, CA, USA), and β-actin (Sigma) were used. Proteins on SDS-PAGE gels were transferred to nitrocellulose filters and stained as described previously [1]. The density of the band with the appropriate molecular mass was determined semi-quantitatively by densitometry using an image analyzing system (Alpha Innotech, San Leandro, CA). The ratio of HSP70 and Bcl-2 and respective β-actin density was used to compare among groups.

### Statistical analyses

All values were expressed as mean ± standard error mean (SEM). Differences within groups were evaluated by paired *t*-test. One-way analysis of variance was used for establishing differences among groups. Intergroup comparisons were made by Duncan's multiple-range test. Differences were regarded as significant if *P *< 0.05 was attained.

## Results

### Effect of PHGG on scavenging O_2_^-.^, H_2_O_2 _and HOCl activity

**Figure 2:**
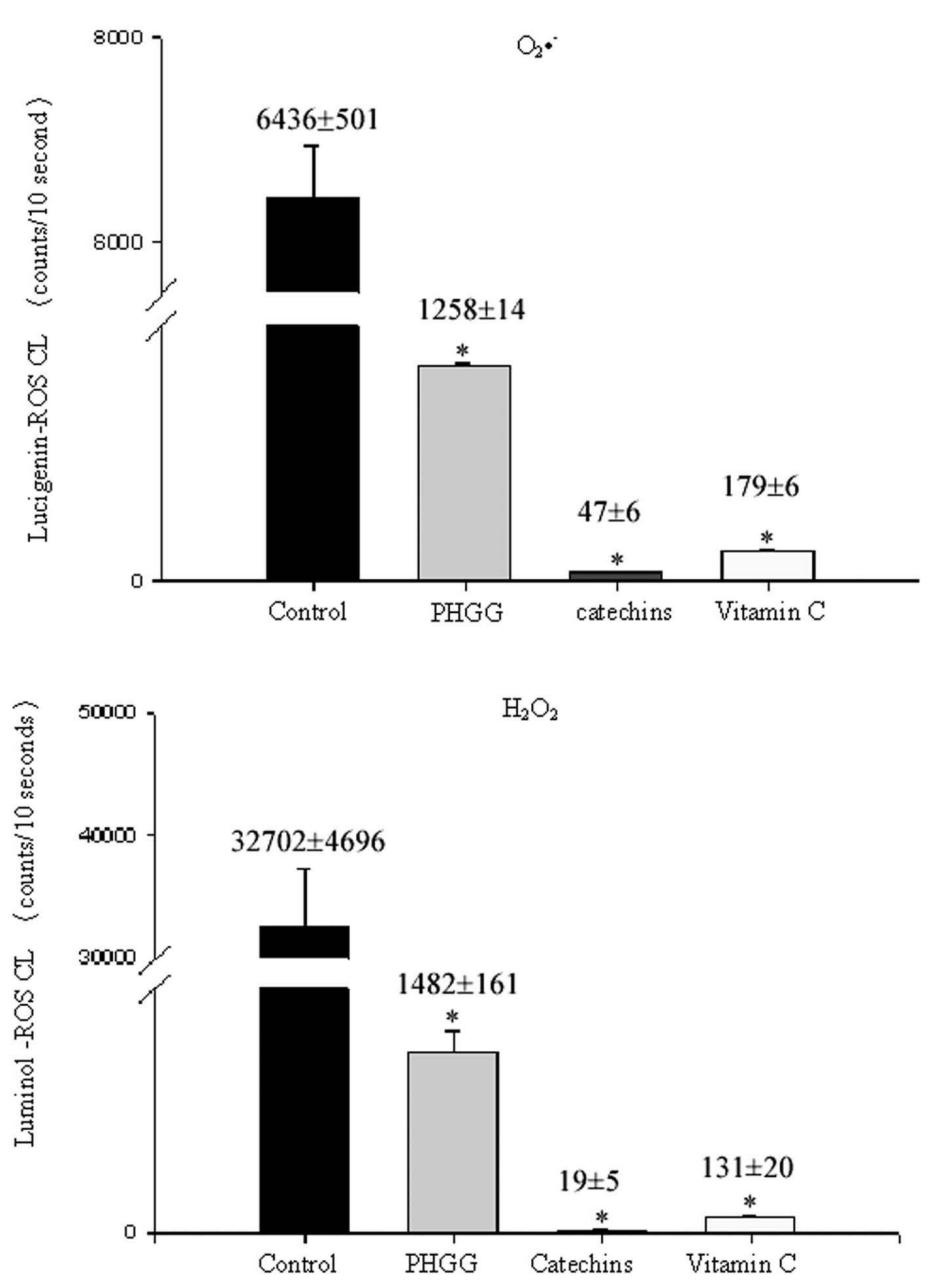
**The antioxidant activity against O_2_•^-^, H_2_O_2_, HOCl in control (distilled water), PHGG, catechins and vitamin C is demonstrated. Catechins, vitamin C and PHGG significantly reduced O_2_•^-^, H_2_O_2_, HOCl activity in an order of catechins > vitamin C > PHGG > control**. Each test was performed 6 times. * *P *< 0.05 when compared to the control value.

We first compared the antioxidant O_2_^-.^, H_2_O_2_, HOCl activity of PHGG in 10 μg/mL. We found that the ability in scavenging O_2_^-.^, H_2_O_2 _and HOCl activity by PHGG, catechins, vitamin C and distilled water was displayed in an order of catechins > vitamin C > PHGG > distilled water as a control value (Figure 2).

### Effect of PHGG on caloric intake and body weight gain

**Figure 3:**
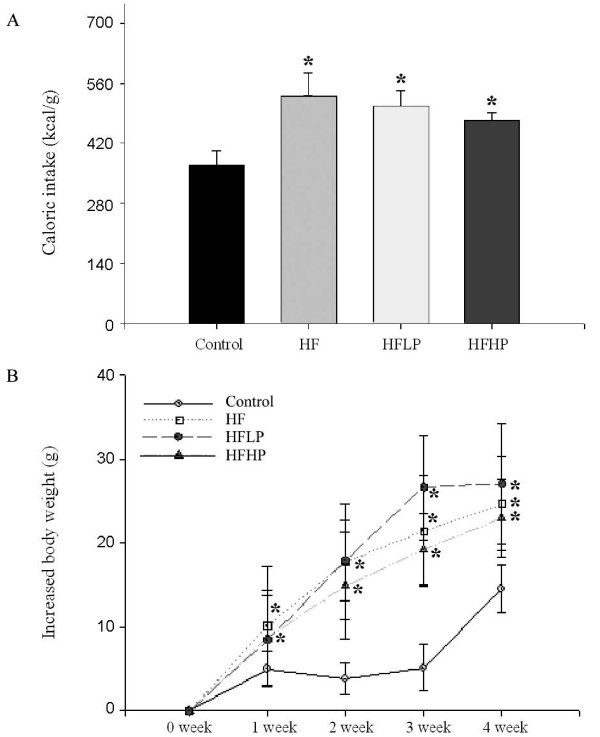
**Effect of PHGG on carolic intake and the increased body weight in the four groups of animals**. Each group was performed in 11 animals. * *P *< 0.05 when compared to the control value or 0 week, respectively.

Our results showed that HF significantly (*P *< 0.05) increased the caloric intake and increased body weight gain in the HF, HFLP and HFHP groups when compared to the control group. Two dosages of PHGG did not significantly decrease caloric intake and growth of animals fed with HF (Figure 3). However, there is a tendency for HFLP and HFHP in the reduction of caloric intake and body weight gain.

### PHGG decreased lipid profiles and oxidative stress

The lipid profiles in the four groups of animals are displayed in the Table 1. We found that four weeks of HF significantly increased TG, T-CHO, HDL, LDL and VLDL, but not LP(a) level with comparison to the control group. Low dose of PHGG significantly reduced HF-enhanced T-CHO and LDL level, whereas high dose of PHGG significantly depressed HF-enhanced TG, T-CHO, LDL and VLDL lelvel.

**Table 1:** Lipid profiles and oxidative stress in the four groups of hamsters.

	Group			
	
Parameters	Control (n = 11)	HF (n = 11)	HFLP (n = 11)	HFHP (n = 11)
TG (mg/dl)	44.5 ± 11.1	98.9 ± 17.4*	88.3 ± 13.5*	47.9 ± 11.1#
T-CHO (mg/dl)	72.9 ± 2.8	110.5 ± 7.1*	89.7 ± 4.7*#	77.2 ± 1.7#
HDL (mg/dl)	46.2 ± 1.9	51.9 ± 1.8*	52.9 ± 2.0*	54.6 ± 0.8*
VLDL (mg/dl)	8.4 ± 2.2	19.6 ± 3.5*	17.7 ± 2.7*	9.6 ± 2.2#
LDL (mg/dl)	18.5 ± 1.3	39.3 ± 5.7*	16.9 ± 3.9#	14.3 ± 1.4*#
LP(a) (mg/ml)	4.8 ± 0.6	6.1 ± 0.5	4.6 ± 0.3	5.8 ± 0.5
MG	89.8 ± 4.8	105.0 ± 4.3*	89.3 ± 4.8#	94.4 ± 8.2#
Dityrosine	1064.1 ± 59.6	1359.4 ± 51.5*	1067.9 ± 45.5#	1002.5 ± 60.0#

Control, feeding with regular diet; High-fat diet (HF), feeding with high-fat diet; High-fat diet + low dose of PHGG (0.25 mg/hamster, HFLP); High-fat diet + high dose of PHGG (1.0 g/hamster, HFHP). Data are shown as mean ± standard error mean. * *P *< 0.05 vs. Control group; # *P *< 0.05 vs. HF group.

On the other hand, oxidative stress indicated by methylguanidine and dityrosine is also significantly elevated in the HF group when compared to the control group. HFLP and HFHP efficiently reduced these oxidative markers when compared to the HF group.

### PHGG attenuated FeCl_3 _induced arterial injury

**Figure 4:**
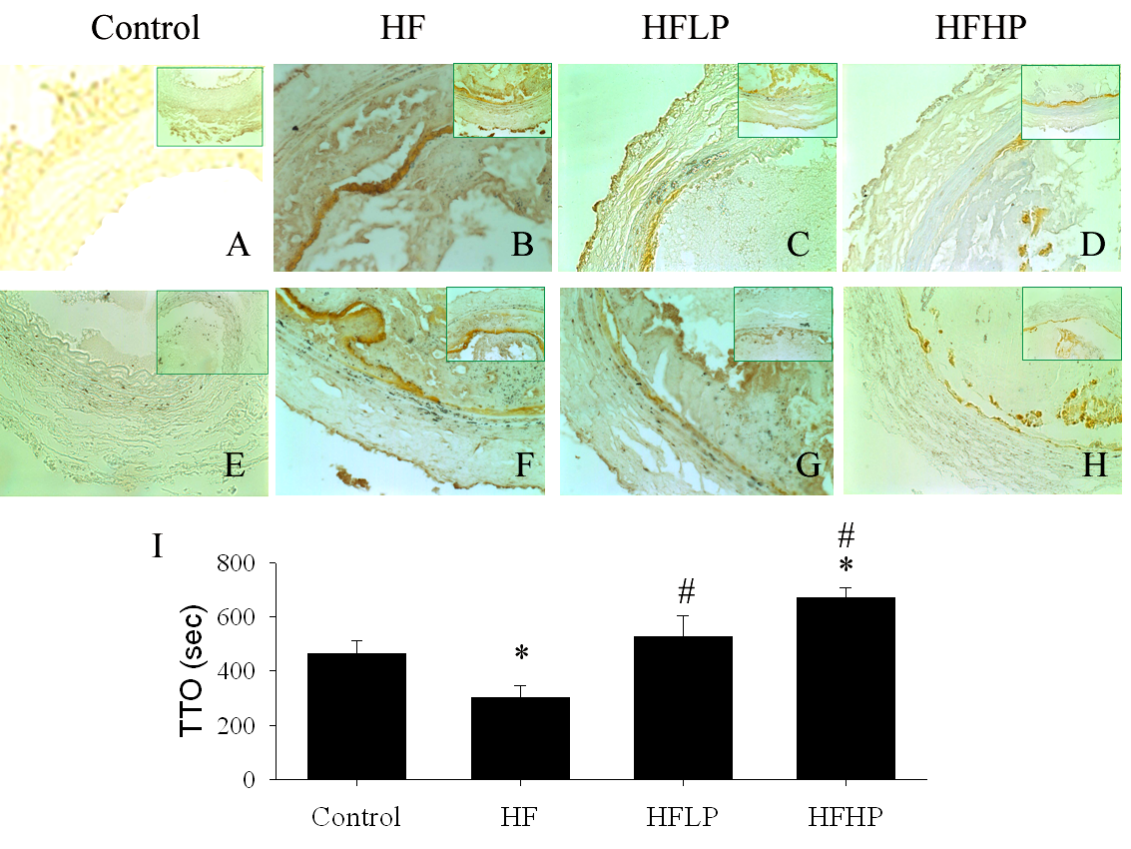
**Effect of PHGG on 30% FeCl_3_-induced 4-HNE, ICAM-1 and TTO in the injuried carotid artery of the hamsters with HF diet**. Oxidative stress indicated by 4-HNE (**A-D**) and ICAM-1 (**E-H**) immunostaining of hamster carotid arterial sections subjected to 150 sec of FeCl_3_-induced arterial injury. Significantly enhanced endothelial ICAM-1 (brown color in **B**) and and 4-HNE (brown color in **F**) are found in HF group subjected to FeCl_3 _treated carotid artery. The amplified diagram in the right corner was displayed. Carotid arterial 4-HNE and ICAM-1 expression were significantly reduced in HFLP and HFHP groups. FeCl_3 _induced time to occlusion (TTO) of carotid arteries obtained from control (n = 11), HF (n = 11), HFLP (n = 11) and HFHP (n = 11) groups is indicated in the I. HF shortened TTO, but was reversed by PHGG treatment. *P < 0.05 vs. control group. # *P *< 0.05 vs. HF group.

At the indicated time of 150 sec-FeCl_3 _stimulation, we observed that enhanced 4-HNE (Figure 4B) and ICAM-1 expression (Figure 4F) was noted in the endothelial site of the damaged carotid artery in the HF group when compared to the control group. We found that FeCl_3 _induced arterial injury evaluated by TTO was 463 ± 51 sec in the control group, 303 ± 45 sec in the HF group, 528 ± 75 sec in the HFLP and 671 ± 36 sec in the HFHP group, respectively (Figure 4I). HF significantly (*P *< 0.05) promoted the arterial thrombosis formation when compared to the control group. Low and high dose of PHGG significantly attenuated FeCl_3 _induced arterial injury by the increased TTO.

### PHGG increased Bcl-2 and HSP-70, but decreased Bax protein expression

**Figure 5:**
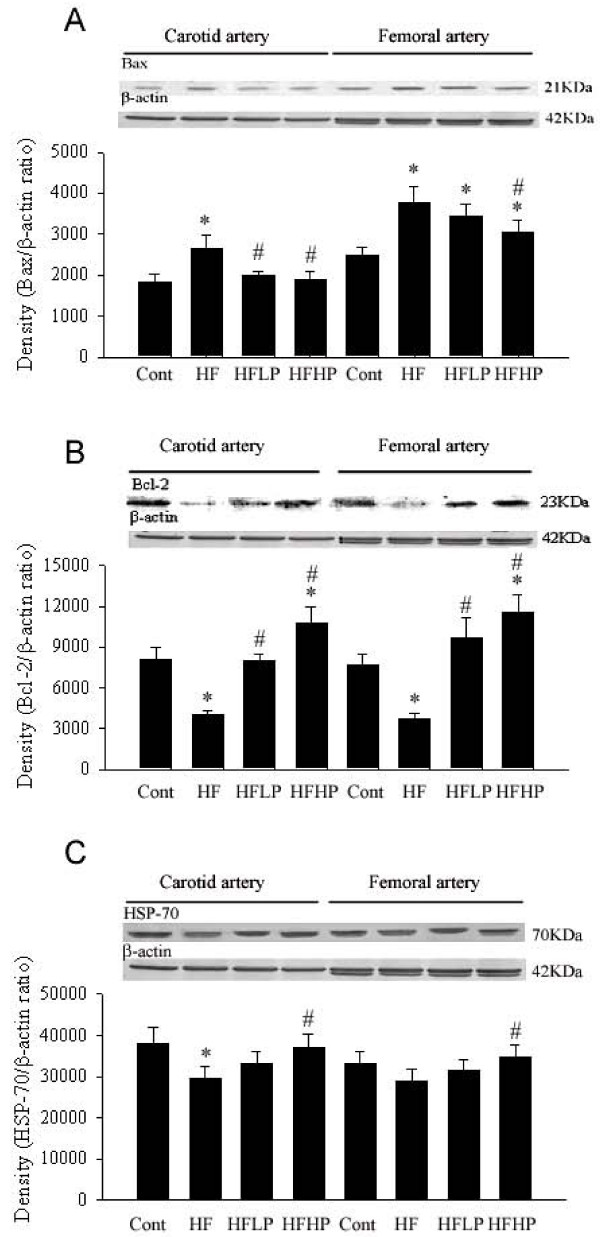
**Effect of low and high dosage of PHGG on Bax, Bcl-2, and HSP-70 expression in the carotid artery and femoral artery of HF fed hamsters**. HF increased Bax and decreased Bcl-2 and HSP-70 expression in the hamster carotid and femoral arteries. HFLP and HFHP treatment decreased Bax and increased Bcl-2 and HSP-70 expression in both carotid and femoral arteries. *P < 0.05 vs. control group. # *P *< 0.05 vs. HF group.

As shown in Figure 5, we found that after four weeks of HF diet significantly decreased the Bcl-2 and HSP-70 and enhanced Bax protein expression in the carotid artery and femoral artery. The enhanced Bax and decreased Bcl-2 expression significantly amplified the Bax/Bcl-2 ratio and possibly contributed to FeCl_3 _induced carotid arterial injury. PHGG treatment preserved the decrease in Bcl-2 and HSP-70 expression and diminished the enhancement in Bax expression in the carotid artery and femoral artery. Also, a further increase in PHGG supplement seems to enhance the Bcl-2 and HSP-70 expression in the carotid and femoral arteries.

## Discussion

We found that an antioxidant O_2_^-.^, H_2_O_2_, HOCl activity was displayed in an order of catechins > vitamin C > PHGG when compared to the water control. The novel biological function of PHGG as a O_2_^-.^, H_2_O_2_, and HOCl scavenger was first demonstrated in the present study. The combined effect of lipid-lowering and antioxidant activity by 4 weeks of PHGG supplement contributes to the reduction of blood lipids and oxidative stress and the delay of arterial thrombus by the upregulation of endogenous antioxidants and downregulation of ICAM-1.

Guar gum with viscous hydrocolloids may reduce the rate of gastric emptying and the diffusion of nutrients to the mucosa of the small intestine and increase cecal output [18]. For example, guar gum changed bile acid pools and intestinal reabsorption to lower cholesterols [19] and decreased trichloroethylene accumulation in the body by reducing trichloroethylene absorption and fat tissue mass [20]. In our study, PHGG supplement seems to have a tendency to depress HF increased caloric intake. However, PHGG supplement didn't significantly decrease body weight gain when compared to the HF group without PHGG supplement. Previous study showed that guar gum in semisolid meal resulted in a more moderate change in blood glucose and positively affected satiety, while general appetite, total energy intake and meal pattern did not differ [21]. Further data also supported there were no significant differences in body weight between control rats and rats fed the guar gum diets [22]. It has been suggested that PHGG with a reduction in viscosity by partial hydrolysis, its efficiency may be greatly diminished [23,24]. Yet, several reports have indicated that PHGG like guar gum efficiently increases bile acid secretion and leads to a cholesterol-lowering effect in rats fed high-fat diet [21,24,25]. The mechanism for an insignificant effect of PHGG on body weight gain requires further investigation in future.

Dietary cholesterol and fat is well known to elicit an accumulation of cholesterol esters and triglycerides in the liver possibly via an enhancement of cholesterol-7α hydroxylase activity for bile acid synthesis, and a suppression of HMG-CoA reductase activity for rate-limiting cholesterol synthesis, contributing to hyperlipidemia [19]. The supplementation of PHGG to lower the intestinal uptake of fat and cholesterol is an interesting strategy to reduce the risk of vascular disease. Our results showed that PHGG significantly decreased blood lipid profiles in the HF fed hamsters. Several mechanisms could be involved. Intake of 3–6% PHGG decreases the bioaccessibility of both fat and cholesterol through the depletion flocculation mechanism to inhibit bile salt induced emulsification and decreases lipolytic activity in the healthy volunteers [25]. PHGG supplement accelerated oxidation of cholesterol toward bile acids accompanied by a marked reduction in hepatic cholesterol and enhanced HMG-CoA reductase activity in the rat [18]. Other notion has also indicated that accelerated cecal bile acid absorption and steroid fecal excretion appear to accompany a greater intestinal bile acid absorption and portal flux to the liver [19]. PHGG can bind the components of micelles in the small intestine: bile acids, but also phospholipids or cholesterol [26]. These mechanisms implicated that PHGG or guar gum contributed to a strong antidyslipidemic potential [23-25]. In our study, low dose of PHGG decreased HF enhanced T-CHO and LDL levels, whereas high dose of PHGG further depressed HF-enhanced TG, T-CHO, LDL and VLDL level in the hamsters fed with HF diets. Both low and high dose of PHGG decreased total cholesterol and LDL. High dose of PHGG exerts more efficient in reduction triglyceride, total cholesterol, LDL, and VLDL level. The present study provides important information that 4 weeks of PHGG supplement can ameliorate HF induced hyperlipidemia.

Guar gum administration combats oxidative stress resulting in a trend to lower oxidative modification of LDL [27]. This data implicate that PHGG obtained from guar gum may have a direct antioxidant activity against oxidative stress. We have used a well-established enhanced CL method for measurement of various types of ROS generation [1-5]. This method has been applied for measurement of ROS production in cultured cells, the whole-blood system, isolated perfused organs, urinary bladder, and kidney *in vivo *[1-5]. Our evidence indicated that the enhanced level of O_2_^-.^, H_2_O_2_, HOCl was significantly depressed by PHGG. In addition, we found that the enhanced antioxidant defense mechanism of Bcl-2 and HSP-70 and the depressed apoptotic/oxidant mechanism of Bax expression were noted after 4 weeks of PHGG supplement. Low and high dose of PHGG supplement for four weeks significantly reduced plasma methylguanidine and dityrosine levels, two oxidative stress markers, in the HF fed hamsters. We also found that FeCl_3 _treatment significantly increased 4-HNE accumulation, a lipid proxidation product, in the endothelial site of carotid artery, indicating oxidative injury existing in the vascular area. PHGG supplement reduced 4-HNE production in the damaged endothelium.

Increased ROS can also trigger the translocation of NF-κB and AP-1 to nucleus and activation of inflammatory cytokines and ICAM-1 that, in turn, contributes to further production of ROS by leukocyte adhesion and inflammation [28]. Enhanced ICAM-1 expression in the endothelial site can increase leukocyte adhesion and platelet aggregation and promoted thrombus formation of the injured vessel [11,15,28]. We found that HF fed animals are associated with the increase of oxidative stress and subsequently promoted FeCl_3 _induced arterial thrombus formation and the impairment of vascular integrity. Our data showed that four weeks of PHGG supplement significantly down regulated ICAM-1 expression in the endothelial site of the FeCl_3 _treated carotid artery and significantly delayed the time of FeCl_3_-induced occlusive arterial thrombus in a dose-dependent manner. The beneficial effect could be due to the synergistic effect of a upregulation in antioxidant Bcl-2 and HSP-70 protein, a suppression of endothelial ICAM-1 protein expression, and a reduction in leucocyte and platelet adhesion, consequently leading to the delay of FeCl_3 _induced thrombus formation in the vascular endothelium of HF fed hamsters.

PHGG supplement decreased cholesterol and lipid levels and oxidative stress and thus has a potential to prevent hypertension and cardiovascular diseases [22,29,30]. After 50 days of PHGG administration, attenuation of urinary protein excretion, total cholesterol level and the morphological changes peculiar to diabetic nephropathy were observed in the rats [16]. Moreover, PHGG can delay the elevation of blood sugar and potentially control the blood sugar in the diabetic patients [31]. Based on these evidences, we implicate that PHGG had a potential in reduction of cardiovascular attack.

In conclusion, dietary supplementation with PHGG in hamsters fed with high-fat diet reduced plasma cholesterol and lipid profiles and increased antioxidant Bcl-2 and HSP-70 protein expression in the vessels. High dose of PHGG was more potent than low dose of PHGG in all these effects. Our study further demonstrated that PHGG inhibit FeCl_3 _enhanced oxidative stress and ICAM-1 expression and delay arterial thrombus formation in hamsters fed with high-fat diet. The clinical implications of these findings are enormous in terms of recommending supplementation of commercial antioxidant diet fibril to patients with vascular disease or those at high risk of developing it.

## Competing interests

The authors declare that they have no competing interests.

## Authors' contributions

DCK, SPH, and CTC conceived the hypothesis, contributed to the design and conduct of the study, conducted the statistical analyses, drafted the manuscript and critically revised manuscript. All authors read and approved the final manuscript.
